# Dr. George H. Reynolds

**Published:** 1912-06

**Authors:** 


Dr. George H. Reynolds, formerly of Delhi, N. Y., died in Niwot, Col., March 21, 1912, of tuberculosis. He was a graduate of Albany, 1891, and was 46 years old.

